# Efficacy of first-line treatments in the elderly and non-elderly patients with advanced epidermal growth factor receptor mutated, non-small cell lung cancer: a network meta-analysis

**DOI:** 10.1186/s12885-022-09592-3

**Published:** 2022-05-07

**Authors:** Ziyi Xu, Chengcheng Liu, Yixiang Zhu, Zihua Zou, Tongji Xie, Puyuan Xing, Le Wang, Junling Li

**Affiliations:** 1 Department of Medical Oncology, National Cancer Center/National Clinical Research Center for Cancer/Cancer Hospital, Chinese Academy of Medical Sciences and Peking Union Medical College, Beijing, 100021 China; 2 Department of Colorectal Surgery and Oncology, The Second Affiliated Hospital, Zhejiang University School of Medicine, Hangzhou, China; 3 Department of Cancer Prevention, Institute of Basic Medicine and Cancer (IBMC), The Cancer Hospital of the University of Chinese Academy of Sciences (Zhejiang Cancer Hospital), Chinese Academy of Sciences, Hangzhou, 310022 Zhejiang China

**Keywords:** NSCLC, *EGFR*-TKIs, Network meta-analysis, Elderly, Non-elderly

## Abstract

**Objective:**

Epidermal growth factor receptor (*EGFR*)-tyrosine kinase inhibitors (TKIs) are the current standard of care for advanced or metastatic non-small cell lung cancer (NSCLC) patients harboring *EGFR* activating mutations. However, the optimal strategy for elderly NSCLC patients is still under debate. This study was designed to explore the optimal first-line regimens by comparing diverse strategies for elderly and non-elderly *EGFR*-mutated NSCLC patients.

**Methods:**

A systematic review was conducted to summarize all available randomized controlled trials (RCTs) from PubMed, EMBASE, Cochrane Central Register of Controlled Trials databases, and international conferences before September 30, 2020. The primary outcome was progression free survival (PFS), and the secondary outcome was overall survival (OS). A network meta-analysis (NMA) was constructed using the Bayesian statistical model to synthesize the survival outcomes of all the treatments.

**Results:**

In total, 12 RCTs were deemed eligible for inclusion with 3779 patients who have received 10 diverse treatments including *EGFR*-TKIs. Results from the Bayesian ranking suggested that osimertinib was most likely to rank the first in overall population and in elderly patients in PFS, with the cumulative probabilities of 42.20% and 31.46%, respectively. In non-elderly group (younger than 65 years old), standard of care (SoC, representing first-generation *EGFR*-TKIs in this NMA) + chemotherapy ranked the first (31.66%). As for OS, SoC + chemotherapy ranked first in all patients (64.33%), patients younger than 65 years old (61.98%), or older than 65 years old (34.45%).

**Conclusion:**

The regimen of osimertinib is associated with the most favorable PFS in elderly advanced *EGFR*-mutated NSCLC patients, while SoC + chemotherapy is the optimal strategy in PFS for non-elderly NSCLC patients harboring *EGFR* activating mutations, and in OS for both elderly and non-elderly *EGFR*-mutated advanced NSCLC patients.

**Trial registration:**

INPLASY protocol 2020100061 10.37766/inplasy2020.20.0061.

**Supplementary Information:**

The online version contains supplementary material available at 10.1186/s12885-022-09592-3.

## Introduction

Lung cancer continues to be the leading cause of cancer-related death worldwide, and around 85% of cases are non-small cell lung cancer (NSCLC) [1]. Most cases are in advanced or metastatic stage at the time of diagnosis, and 47% of patients with lung cancer are 70 years of age or older [2]. A proportion of elderly people are poor in health and are often with disease in multiple organs and systems, and thus a discussion of cancer management in elderly patients is timely. The standard treatment for elderly cancer patients is still under debate, mainly because elderly patients are often excluded from or underrepresented in clinical trials. In the recent years, due to the increasing number of elderly patients likely owing to an increase in life expectancy, a greater attention has been given to this population, and more elderly patients have been included in clinical trials for subgroup analysis. Generally, 65 years of age is commonly used as a reference point in clinical trials for lung cancer.

Epidermal growth factor receptor (*EGFR*)-tyrosine kinase inhibitors (TKIs) are the standard first-line therapy for NSCLC patients harboring activating *EGFR* mutations [3, 4]. Results have shown that orally taken TKIs not only bring survival benefit to specific population, but have also been well tolerated compared to traditional chemotherapy [5, 6]. There are diverse kinds of *EGFR*-TKIs in clinicial use currently, and studies of these drugs have included populations with many different baseline characteristics, including age. However, to the best of our knowledge, no prospective trials have been conducted specifically in elderly patients. Some trials accrued an older cohort, such as the EURTAC trial [7] and the BR.21 study [8], which indicated that *EGFR*-TKIs improved survival but yielded a worse adverse event profile in elderly patients. In the older cohort of the BR.21 study, elderly patients had significantly more overall and severe (grade 3 and 4) toxicity compared with young patients, and were more likely to cease treatment due to treatment-related adverse events [9].

Therefore, this study was designed to explore the optimal first-line regimens by comparing diverse strategies for elderly and non-elderly *EGFR*-mutated NSCLC patients by comparing the efficacy of diverse first-line treatments with gefitinib, erlotinib, afatinib, dacomitinib, and osimertinib, either as monotherapy or combining with other anti-tumor therapy such as chemotherapy and anti-angiogenic agents, for elderly and non-elderly patients harboring activating *EGFR* mutations (exon 19 deletion or exon 21 L858R mutation) NSCLC by conducting a network meta-analysis (NMA) of all available evidence in the literature, so as to explore the optimal treatment for both population. By synthesizing direct and indirect evidence, NMAs could compare a number of interventions with diverse comparators simultaneously, which is applied under the circumstance of lacking head-to-head trial data [10].

## Methods

### Participants and methods

The Preferred Reporting Items for Systematic Reviews and Meta-Analyses (PRISMA) statement [11] statement was utilized as a reporting guideline. The registration of this protocol can be found on the International Platform of Registered Systematic Review and Meta-analysis Protocols (INPLASY), which is available https://inplasy.com/inplasy-2020-10-0061/.

### Searching strategies

This systematic literature review was designed to assess the efficacy of diverse first-line therapy for elderly and non-elderly NSCLC patients harboring activating *EGFR* mutations from clinical trials. Related publications were searched and screened in PubMed, EMBASE and the Cochrane Central Register of Controlled Trials databases before September 30, 2020. The combinations of Medical Subject Headings (MESH) terms (ie, “NSCLC”, “EGFR”, “TKI”, “PFS”, “OS” and “Randomized Controlled Trial”, “RCT”) and free text words were used for searching. Supplemented literature search was conducted for included potential studies in meeting libraries of American Society of Clinical Oncology (ASCO), European Society of Medical Oncology (ESMO), European Cancer Conference (ECC), and World Conference (WCLC) on Lung Cancer. Details were presented in previous study [12]. The searching strategies were shown in Table S1, S2, and S3.

### Outcome definition

The primary outcome was progression free survival (PFS), and the secondary outcome was overall survival (OS).

### Eligibility and study selection

Eligible studies were stage IIb/III randomized controlled trials (RCTs) that compared that efficacy of first-line strategies with a single *EGFR*-TKI or the combination therapy with *EGFR*-TKI to another TKI or to TKI monotherapy or to standard platinum-based chemotherapy for stage IIIB/IV NSCLC patients harboring activating *EGFR* mutation.

Inclusion criteria were defined as followings: 1) Types of participants: Advanced NSCLC patients with activating *EGFR* mutation; 2) Types of interventions: *EGFR*-TKIs with or without anti-angiogenic agent; 3) Types of controls: *EGFR*-TKIs or chemotherapy; 4) Types of outcomes: OS, PFS; 5) Types of studies: RCTs.

### Data extraction and quality assessment

Based on the inclusion and exclusion criteria listed above, eligible studies were initially screened by reading the titles and abstracts of all identified record. Paper evaluations and data extraction were performed by two members, Z. and YZ independently, and cases of discrepancy was resolved by a third expert, JL The risk of biased were further assessed with the Cochrane risk-of-bias tool [13]. Snowball search for reference lists of published systematic reviews and meta-analyses were reviewed.

A pro-form-designed by review working group for data extraction was as followings: 1) Basic information: name of study, year of publication, country. 2) Trials design: type of design, characteristics of participants, sample size, treatment strategies in the intervention and the control group. 3) Clinical outcomes: PFS and OS data.

### Data analyses

The log hazard ratios (HR) of PFS and OS were used to perform NMAs. The network plots were drawn to present interactions of various treatment options by using Stata software (version 15.0) [14]. Heterogeneity across included studies was assessed by Q test and I^2^, and I^2^ values < 25%, 25% to 50%, and ≥ 50% indicate low, moderate, and high heterogeneity, respectively [15].

Bayesian network meta-analysis was applied in this NMA by using a Markov Chain Monte Carlo simulation technique. The “gemtc” and “rjags” packages in R software (version 3.6.3) were used and the random effect model was chosen by using the odds ratio (OR) and 95% CI to compare the intervention measures [16].

Based on previous researches, the fixed model in the registration was supposed to be used for NMA, while the random effects model was used given that high heterogeneities existed. For PFS in all patients, high heterogeneities were detected between Chemotherapy and Afatinib (I^2^ = 78.2%), standard of care (SoC, representing first-generation *EGFR*-TKIs in this NMA) and Chemotherapy (I^2^ = 98.9%), SoC and Chemotherapy (I^2^ = 98.9%). For PFS in patients below 65, comparisons from Chemotherapy and Afatinib (I^2^ = 61.9%), SoC and Afatinib (I^2^ = 79.8%), SoC and Chemotherapy (I^2^ = 74.4%) showed high heterogeneities. For PFS in patients above 65, high heterogeneities were found between Chemotherapy and Afatinib (I^2^ = 81.5%), SoC and Afatinib (I^2^ = 54.5%). Similar high heterogeneities were found for OS among all patients between SoC and Afatinib (I^2^ = 65.7%). For OS in patients older than 65 years, comparisons of Chemotherapy and Afatinib (I^2^ = 84.9%), SoC and Afatinib (I^2^ = 78.0%), SoC and Chemotherapy (I^2^ = 75.1%) showed high heterogeneities.

Posterior sampling was performed using Markov Chain Monte Carlo. Four chains of initial values were generated to fit the model with 10 000 sample iterations. Each chain was set with 5 000 burn-ins and a thinning interval of 5. Convergence was evaluated through visual inspection and the density plot (Figure S1) [17]. Under the Bayesian framework, the NMA estimated the overall rankings of treatments.

Transitivity and consistency were two key assumptions in support of the NMA. For the transitivity, trials with strict patient allocation were identified and included, and similar condition for evaluated treatment options was optimized. For inconsistency, comparing the fit of consistency and inconsistency models were evaluated [18].

## Results

### Characteristics

**Fig. 1:**
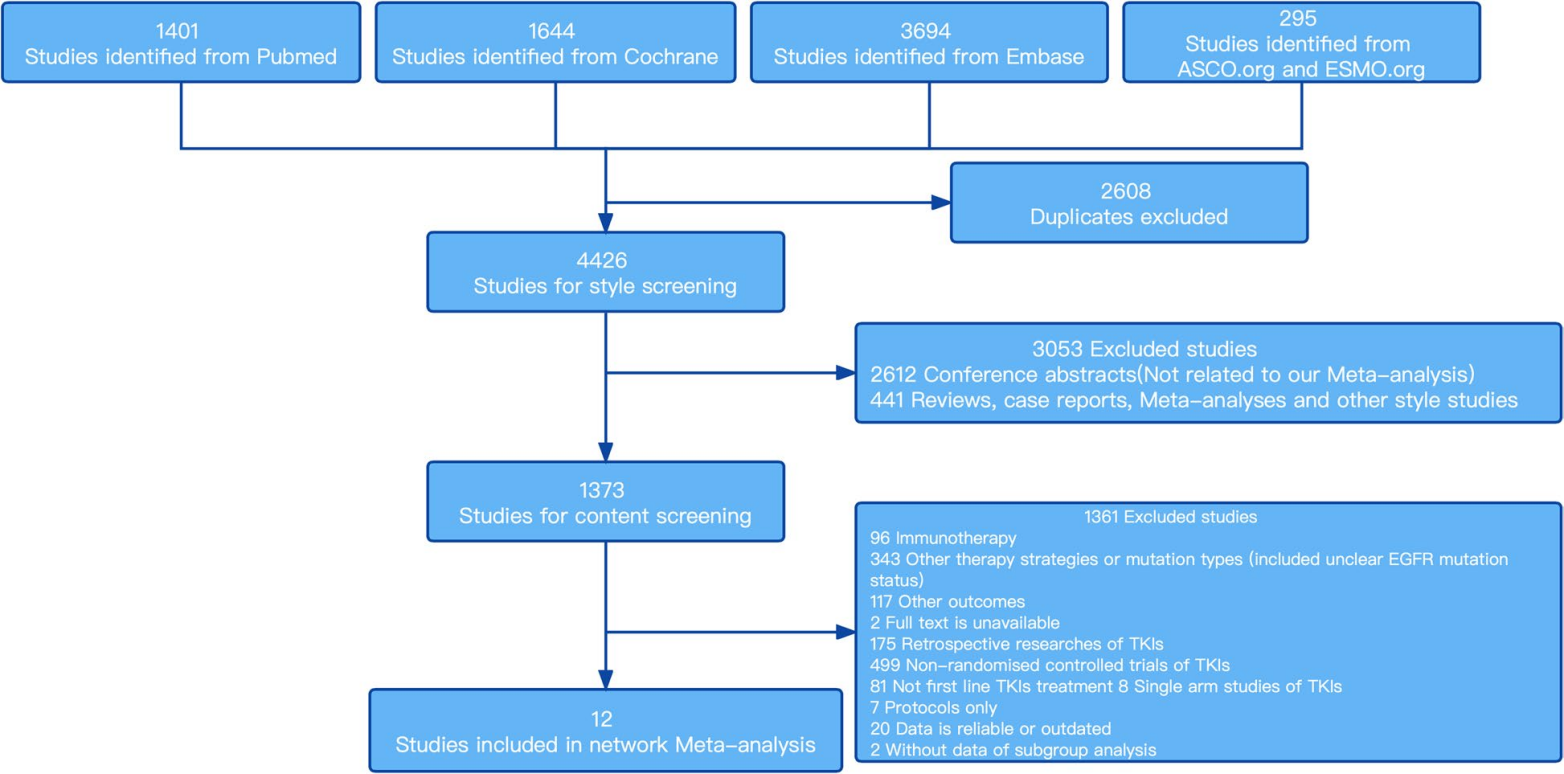
Study selection

Electronic search in the database resulted in 7034 records, from which 2608 internal and external duplicates were excluded. After style screening and content screening, 12 RCTs were deemed eligible for inclusion with a total of 3779 patients to receive 10 different treatments including *EGFR*-TKIs (erlotnib, gefitinib, afatinib, dacomitinib, and osimertinib), pemetrexed-based chemotherapy, pemetrexed-free chemotherapy, and combination therapy(erlotinib plus ramucirumab, gefitinib plus apatinib, and gefitinib plus pemetrexed-based chemotherapy), with the subgroup analysis performed in patients below 65 and above 65 years old. Studies like the NEJ026 and the ARTEMIS study were excluded from this analysis, for the subgroup analysis was performed in patients below 75 and above 75 years old. The flow chart was presented in Fig. 1. The main characteristics of all studies were shown in Table 1.

**Table 1:** The baseline characteristics of all included studies in the NMA for advanced *EGFR*-mutated NSCLC patients

Study (phase, ethnicity)	Sample size (No.);median age	Female (%)	Age		Intervention arm	Control arm	Reported outcomes
			< 65	≥ 65			
EURTAC, 2012 (III, non-Asian)	86/87; 65/65	64/62	NG	NG	Erlotinib	Chemotherapy (cisplatin + docetaxel)	Progression-free survival
OPTIMAL, 2011 (III, Asian)	82/72; 57/59	59/60	63/51	19/21	Erlotinib	Chemotherapy (gemictabine + caboplatin)	Progression-free survival, over-all survival
FLAURA, 2018 (III, multiple)	279/277; 64/64	64/62	NG	NG	Osimertinib	Gefitinib/Erlotinib	Progression-free survival, over-all survival
RELAY, 2019 (III, multiple)	224/225; 65/64	63/63	102/114	122/111	Erlotinib + ramucirumab	Erlotinib	Progression-free survival
ACTIVE, 2020 (III, Asian)	157/156; 57/60	58/60.3	117/113	40/43	Gefitinib + apatini	Gefitinib	Progression-free survival
JMIT, 2016/2019 (II, Asian)	126/65; 62/62	65.1/63.1	79/43	47/22	Gefitinib + pemetrexed	Gefitinib	Progression-free survival
NEJ009, 2020 (III, Asian)	170/172; NG	67.1/62.8	NG	NG	Gefitinib + pemetrexed + + carboplatin	Gefitinib	Progression-free survival, over-all survival
Han et al., 2017/2020 (II, Asian)	40/41; NG	62.5/56.1	27/27	13/14	Gefitinib + pemetrexed + carboplatin	Gefitinib	Progression-free survival
ARCHER1050, 2017/2018 (III, multiple)	227/225; 62/61	64.3/55.6	133/140	94/85	Dacomitinib	Gefitinib	Progression-free survival, over-all survival
LUX-LUNG 3, 2013/2015 (III, multiple)	230/115; 61.5/61	63.9/67	NG	NG	Afatinib	Chemotherapy (pemetrexed + cisplatin)	Progression-free survival, over-all survival
LUX-LUNG 6, 2014/2015 (III, Asian)	242/122; 58/58	64/68	NG	NG	Afatinib	Chemotherapy (gemcitabine + cisplatin)	Progression-free survival, over-all survival
LUX-LUNG 7, 2016/2017 (IIb, multiple)	160/159; 63/63	56.9/66.7	NG	NG	Afatinib	Gefitinib	Progression-free survival, over-all survival

Network meta-analysis (*NMA*); epidermal growth factor receptor (*EGFR*); non small-cell lung cancer (*NSCLC*)

### Risk of bias

The risk of bias for included studies was assessed according to the recommendation of Cochrane Reviewers’ handbook. All studies were considered to be at low to medium bias risk.

**Fig. 2:**
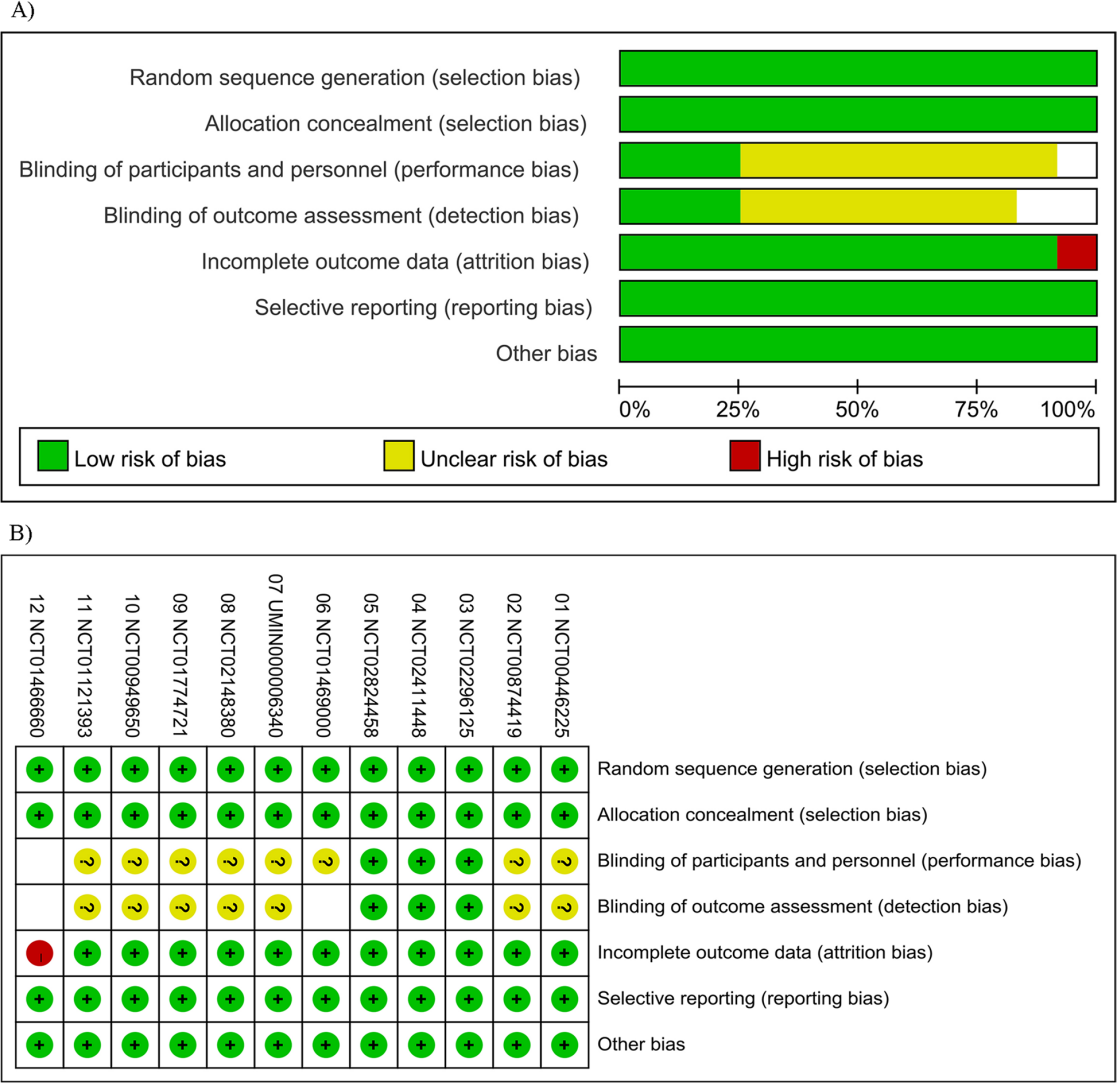
Summary of risk of bias assessment **A**). Risk of bias assessment: overall risk of bias for all included trials. **B**). Risk of bias summary: overall risk of bias for all included trials

Detailed results of the analysis of risk of bias in individual studies for the quality evaluation of included literature were listed in Fig. 2. Most studies had no serious census data, except the trial NCT01466660 [19, 20] with high bias in completeness, for the reason that 34 patients who had no ethnic origin recorded were included in this trial [19]. However, these 34 patients were reported as non-Asian in the updated OS reports of the trial [20]. Although the ethnic information of patients was not analyzed here in our study, the bias of the trial NCT01466660 adversely affects the reliability of its results. Besides, most studies had no statement on the method for blinding in the intervention and outcome.

### Network Meta-analysis

#### Progression free survival: overall and age-specific results

**Fig. 3:**
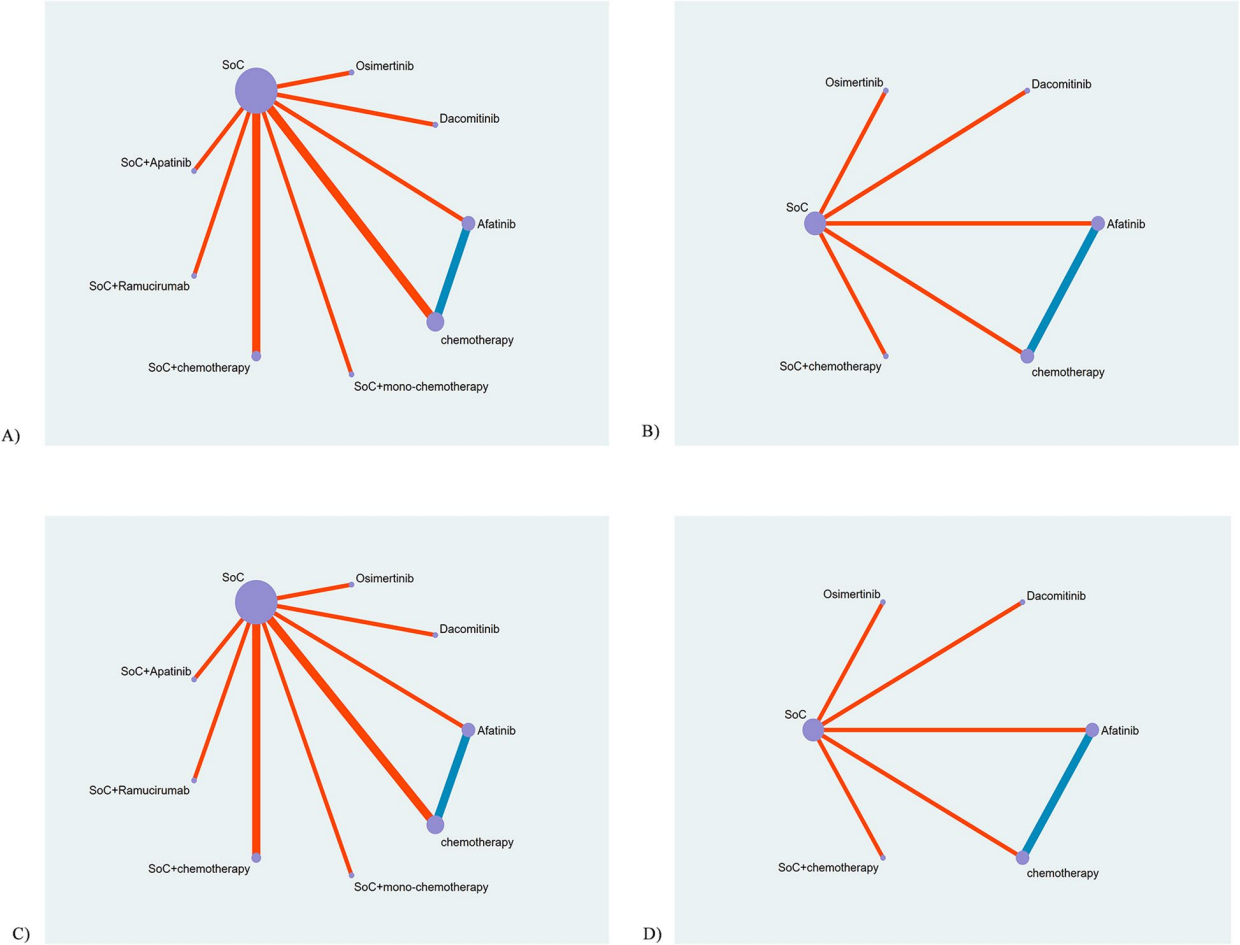
Network diagrams of comparisons on different outcomes of treatments in different race groups of patients with non-small cell lung cancer (NSCLC). **A**) Comparisons for progression free survival on patients below 65 years old. **B**) Comparisons for overall survival on patients below 65 years old. **C**) Comparisons for progression free survival on patients over 65 years old. **D**) Comparisons for overall survival on patients over 65 years old

The NMA included all treatments for PFS, and 10 studies with OS outcome. In patients below 65 years, PFS was reported in all identified literature (Fig. 3A), while OS data was reported in 7 literature (Fig. 3B). In patients above 65 years old, 12 studies and 7 studies reported the PFS (Fig. 3C) and OS (Fig. 3D), respectively.

**Fig. 4:**
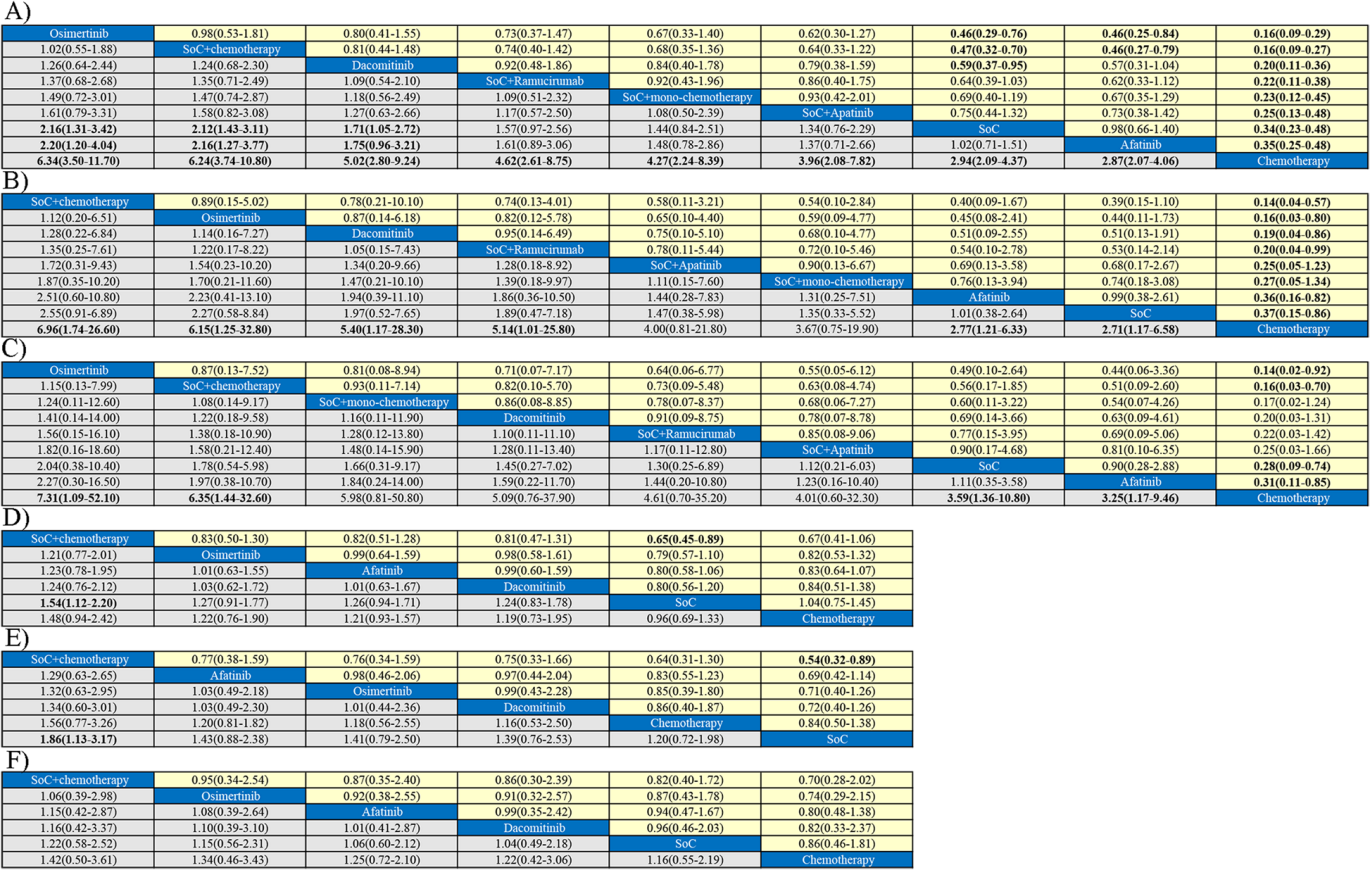
Pooled estimates of the network meta-analysis. Data in each cell are hazard or odds ratios (95% credible intervals) for the comparison of row-defining treatment versus column-defining treatment. Hazard ratios less than 1 and odds ratios more than 1 favor row-defining treatment. Significant results are in bold. PFS: progression free survival, OS: overall survival. **A**) Pooled odds ratios (95% credible intervals) for PFS in all patients. **B**) Pooled odds ratios (95% credible intervals) PFS in patients aged below 65 years. **C**) Pooled odds ratios (95% credible intervals) PFS in patients aged over 65 years. **D**) Pooled odds ratios (95% credible intervals) OS in all patients. **E**) Pooled odds ratios (95% credible intervals) OS in patients aged below 65 years. **F**) Pooled odds ratios (95% credible intervals) OS in patients aged over 65 years

As for PFS for all patients (Fig. 4A), Osimertinib showed superior efficacy than SoC (hazard ratio: 0.46, 95% credible interval: 0.29–0.76), Afatinib (0.46, 0.25–0.84) and Chemotherapy (0.16, 0.09–0.29). Similar phenomenon could be found for SoC + chemotherapy, better efficacy could be found than SoC (0.47, 0.32–0.70), Afatinib (0.46, 0.27–0.79) and Chemotherapy (0.16, 0.07–0.27). Except for Osimertinib, SoC + chemotherapy, Chemotherapy had inferior efficacy in PFS for all patients, than Dacomitinib (0.20, 0.11–0.36), SoC + Ramucirumab (0.22, 0.11–0.38), SoC + mono-chemotherapy (0.23, 0.12–0.45), SoC + Apatinib (0.25, 0.13–0.48), SoC (0.34, 0.23–0.48), and Afatinib (0.35, 0.25–0.48).

For PFS in patients below 65 years old (Fig. 4B), Chemotherapy had worse efficacy than SoC + chemotherapy (0.14, 0.04–0.57), Osimertinib (0.16, 0.03–0.80), Dacomitinib (0.19, 0.04–0.86), SoC + Ramucirumab (0.20, 0.04–0.99), Afatinib (0.36, 0.16–0.82), and SoC (0.37, 0.15–0.86). For other comparisons, no obvious differences was found for other comparisons.

For PFS in patients over 65 years old (Fig. 4C), Chemotherapy was inferior than Osimertinib (0.14, 0.02–0.92), SoC + chemotherapy (0.16, 0.03–0.70), SoC (0.28, 0.09–0.74), and Afatinib (0.31, 0.11–0.85). 

#### Overall survival: overall and age-specific results

In terms of overall survival for all included patients (Fig. 4D), SoC plus chemotherapy lead to better efficacy than SoC alone (0.65, 0.45–0.89). No significant differences could be found for other comparisons. When we limited to patients below 65 years old (Fig. 4E), similar results could be found as well. SoC alone had inferior efficacy than SoC plus chemotherapy (0.65, 0.45–0.89). As for patients above 65 years old (Fig. 4F), no obvious differences were shown from other comparisons.

### Rank probabilities

**Fig. 5:**
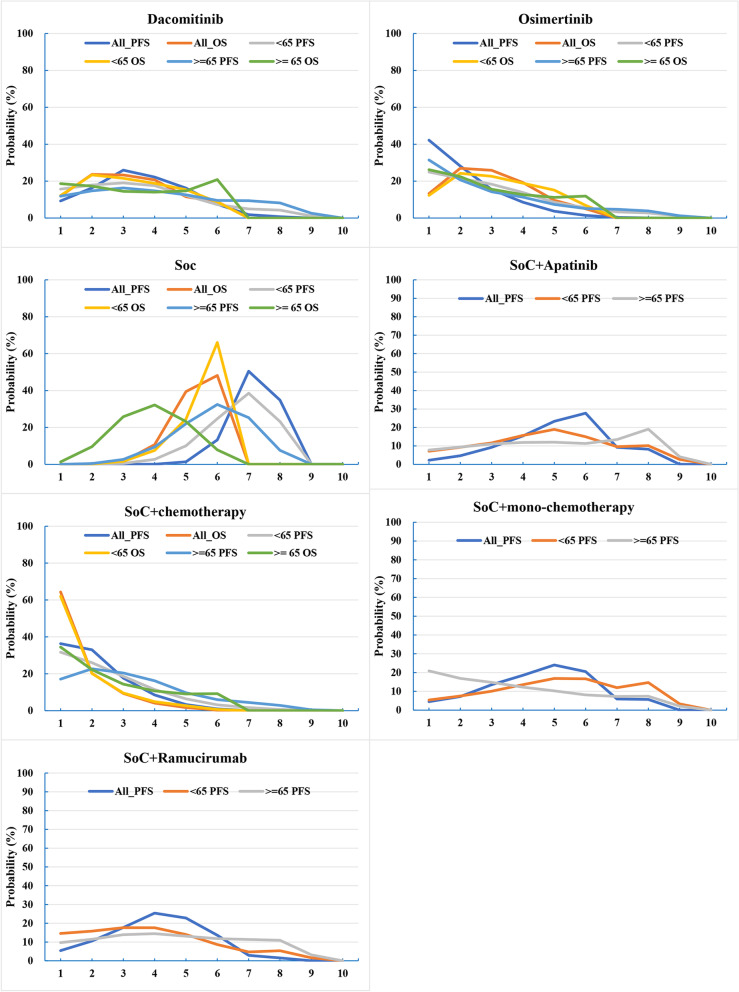
Bayesian ranking profiles of comparable treatments on efficacy for patients with non-small cell lung cancer. Profiles indicate the probability of each comparable treatment being ranked from first to last on progression free survival (PFS), overall survival (OS)

Bayesian ranking profiles of evaluated treatments in different populations were shown in Fig. 5. The Bayesian ranking results were almost in line with the pooled analysis using hazard and odds ratios. In terms of PFS, no matter in all patients or in patients older than 65 years old, osimertinib yielded the best benefit, with 42.20% and 31.46% of cumulative probabilities, respectively. In patients younger than 65 years old, SoC + chemotherapy ranked the first (31.66%). As for OS, SoC + chemotherapy ranked first in all patients (64.33%), in patients younger than 65 years old (61.98%), or older than 65 years old (34.45%).

### Inconsistency assessment

The fit of the consistency model was similar or better than that of inconsistency model (Table S4). No significant differences in comparisons were found between direct and indirect estimates could be found from the node splitting analysis (*P* > 0.05) (Table S5).

## Discussion

*EGFR*-TKIs have been the current standard of care for advanced or metastatic *EGFR*-mutated NSCLC patients [5, 7]. The combination strategy of *EGFR*-TKIs and cytotoxic agents or anti-angiogenic agents has also shown benefit in controlling the disease and prolonging the survival [21, 22]. Although the diverse therapeutic strategies of TKI monotherapy or combination therapy with TKI were feasible for patients of different ages, the decision on anti-tumor strategy is still under debate in elderly NSCLC patients, as no data has been reported from prospective trails. Since more head-to-head clinical trials have been available, we conducted this NMA to compare the efficacy of several *EGFR*-TKIs including first-generation *EGFR*-TKIs, afatinib, dacomitinib, osimertinib, pemetrexed-based or pemetrexed-free chemotherapy, and the combination of *EGFR*-TKIs with chemotherapy or anti-angiogenic agents, in elderly and non-elderly NSCLC patients harboring activating *EGFR* mutations. To our knowledge, this is the largest meta-analysis that evaluated the optimal therapeutic choice of patients with advanced *EGFR*-mutated NSCLC, and also is the first one to identify the optimal therapeutic choice for elderly and non-elderly patients.

Results have indicated that the addition of chemotherapy to SoC was significantly more effective in terms of PFS in subgroups < 65 years old, and has also shown a better PFS compared to other standard care. For elderly group with ≥ 65 years old, osimertinib was superior in terms of PFS, and has also shown a better PFS compared to other standard care. A previous NMA has compared all first-line treatments for advanced *EGFR*-mutated NSCLC patients, and found that gefitinib + pemetrexed-based chemotherapy and osimertinib might be the optimal therapy with the greatest benefits in PFS and OS [23]. Data from our study was consistent with the results, suggesting that osimertinib was the most favorable regimen in terms of PFS in all advanced *EGFR*-mutated NSCLC patients and also in elderly patients who were ≥ 65 years old, and a combination of SoC and pemetrexed based chemotherapy was superior compared to other regimen in non-elderly patients who were < 65 years old. Since the subsequent therapy was not taken into account in the study, this would considerably affect the basis for OS.

Osimertinib prolongs PFS and OS in selected NSCLC patients [4, 24], irreversibly inhibiting both *EGFR* sensitizing mutations and *EGFR* exon 20 T790M mutation [25]. *EGFR* T790M mutation is an acquired mutation that accounts for approximately 50% of resistance after first- or second-generation *EGFR*-TKIs [26, 27]. In the FLAURA study that comparing the efficacy of osimertinib and first-generation *EGFR*-TKIs in the first-line setting of advanced *EGFR*-mutated NSCLC, osimertinib showed improvement in both the median PFS (18.9 *vs.* 10.2 months, *P* < 0.001) [4] and the OS (38.6 *vs.* 31.8 months, *P* = 0.046) [24]. In our study, osimertinib was superior in terms of PFS, and has also shown a better PFS compared to other standard care in all advanced NSCLC patients. Moreover, osimertinib is orally-taken, which is convenient for the elderly patients and their caregivers and would promote the quality of life. Several reports have indicated that *EGFR*-TKIs are favorable in safety profile in elderly NSCLC patients [28, 29], with the same efficacy demonstrated in younger NSCLC patients. Although no significant difference was found in osimertinib and other strategy in terms of OS according to our results, osimertinib seems to be a beneficial and also a safe and feasible treatment for elderly patients.

The majority of patients would develop resistance to first- and second-generation *EGFR*-TKIs after a median duration of 9–13 months [7, 30]. Scientists have investigated the combination strategy of *EGFR*-TKIs and other anti-tumor strategy in order to delay drug resistance. For example, the JMIT study has reported that pemetrexed plus gefitinib achieved a significantly longer PFS [31] and a numerically higher OS rate [32] compared to gefitinib alone as first-line therapy for *EGFR*-mutated NSCLC patients. The NEJ009 study has also demonstrated that the concurrent strategy of gefitinib and pemetrexed plus carboplatin was superior in PFS and OS compared to gefinitib monotherapy, although no significant difference was found in PFS2 considering the influence of the postprogressive disease period [21]. The benefit from the combination of first-generation *EGFR*-TKIs and chemotherapy might be attributed to the synergistic effect with *EGFR*-TKIs on the tumor growth of NSCLC [33]. Also, according to preclinical experiments, gefitinib could cause a dose-dependent reversal of resistance to chemotherapy in NSCLC cell line [34]. In our study, SoC + chemotherapy ranked the best strategy than chemotherapy for non-elderly patients, surpassing osimertinib. For elderly patients, however, SoC + chemotherapy ranked the second-best treatment, surpassed by osimertinib. In terms of OS, SoC plus chemotherapy lead to better efficacy in not only all NSCLC patients, but also in elder patients and non-elderly patients harboring sensitizing *EGFR* mutations.

There are several limitations in this study. First, although our NMA suggests that first-generation *EGFR*-TKIs plus chemotherapy is the most favorable strategy for non-elderly advanced *EGFR*-mutated NSCLC patients, no RCTs have compared the efficacy of combing second or third generation *EGFR*-TKIs and chemotherapy to other anti-tumor treatments. Therefore, it is still worth of exploring on the efficacy of the combination therapy of chemotherapy and second or third generation *EGFR*-TKIs. Second, since the heterogeneity might be influenced by the complexity of subsequent treatment options in different trials with the OS as the endpoint for assessing the efficacy, PFS was taken as the primary endpoint in this NMA instead of OS. Third, although 65 years of age was chosen as a reference point in most clinical trials for lung cancer, subgroup analysis was performed in patients below and above 75 years old in some studies, which were excluded from this NMA. It still requires further exploration on the optimal strategies for the elderly 75 year-old patients in future studies. Forth, since the subgroup analysis of adverse events was not performed in most RCTs, in which the comparison was only made between the experimental group and the control group, this study failed to analyze the toxicity of each strategy for elderly patients. The safety profile of treatments for elderly patients is of great concern in clinical practice, and should be considered in future study design.

## Conclusion

The regimen of osimertinib is associated with the most favorable PFS in elderly advanced *EGFR*-mutated NSCLC patients, while SoC + chemotherapy is the optimal strategy in PFS for non-elderly NSCLC patients harboring *EGFR* activating mutations, and in OS for both elderly and non-elderly *EGFR*-mutated advanced NSCLC patients.

## Supplementary Information

**Additional file 1:** **Table S1. **Literature search criteria for PubMed. **Table S2. **Literature search criteria for Embase. **Table S3. **Literature search criteria for Cochrane. **Table S4. **Comparisions of the fit ofconsistency and inconsistency models using deviance information criteria (DIC). **Table S5. **Node-splitting analysis ofinconsistency.All P value was below 0.05, indicatingno significant inconsistencies between the direct effect and indirect effects. **Figure S1. **Density plot forprogression-free survival (PFS) and overall survival (OS) in all includedpatients, in elderly patients and in non-elderly patients.

**Additional file 2.**

## Data Availability

All data generated or analyzed during this study are included within this article (and its supplementary files).

## References

[1] Morgensztern D, Ng SH, Gao F, Govindan R (2010). Trends in stage distribution for patients with non-small cell lung cancer: a National Cancer Database survey. J Thorac Oncol.

[2] Owonikoko TK, Ragin CC, Belani CP (2007). Lung cancer in elderly patients: an analysis of the surveillance, epidemiology, and end results database. J Clin Oncol.

[3] Mok TS, Wu YL, Thongprasert S, et al. Gefitinib or carboplatin-paclitaxel in pulmonary adenocarcinoma. New Engl J Med. 2009;361:947–57. 10.1056/NEJMoa0810699 19692680

[4] Soria JC, Ohe Y, Vansteenkiste J (2018). Osimertinib in Untreated EGFR-Mutated Advanced Non-Small-Cell Lung Cancer. N Engl J Med.

[5] Mitsudomi T, Morita S, Yatabe Y (2010). Gefitinib versus cisplatin plus docetaxel in patients with non-small-cell lung cancer harbouring mutations of the epidermal growth factor receptor (WJTOG3405): an open label, randomised phase 3 trial. Lancet Oncol.

[6] Wu YL, Zhou C, Hu CP (2014). Afatinib versus cisplatin plus gemcitabine for first-line treatment of Asian patients with advanced non-small-cell lung cancer harbouring EGFR mutations (LUX-Lung 6): an open-label, randomised phase 3 trial. Lancet Oncol.

[7] Rosell R, Carcereny E, Gervais R (2012). Erlotinib versus standard chemotherapy as first-line treatment for European patients with advanced EGFR mutation-positive non-small-cell lung cancer (EURTAC): a multicentre, open-label, randomised phase 3 trial. Lancet Oncol.

[8] Shepherd FA, Rodrigues Pereira J, Ciuleanu T (2005). Erlotinib in previously treated non-small-cell lung cancer. N Engl J Med.

[9] Wheatley-Price P, Ding K, Seymour L, Clark GM, Shepherd FA (2008). Erlotinib for advanced non-small-cell lung cancer in the elderly: an analysis of the National Cancer Institute of Canada Clinical Trials Group Study BR.21. J Clin Oncol.

[10] Jansen JP, Fleurence R, Devine B (2011). Interpreting indirect treatment comparisons and network meta-analysis for health-care decision making: report of the ISPOR Task Force on Indirect Treatment Comparisons Good Research Practices: part 1. Value Health.

[11] Knobloch K, Yoon U, Vogt PM (2011). Preferred reporting items for systematic reviews and meta-analyses (PRISMA) statement and publication bias. J Craniomaxillofac Surg.

[12] Xie T, Zou Z, Liu C (2021). Front-Line Therapy in EGFR Exon 19 Deletion and 21 Leu858Arg Mutations in Advanced Non-Small Cell Lung Cancer: A Network Meta-Analysis. Evid Based Complement Alternat Med.

[13] Higgins JP, Thomas J, Chandler J, et al. Cochrane handbook for systematic reviews of interventions version 5.0.1. Wiley; 2019. 10.1002/14651858.ED000142 PMC10284251 31643080

[14] Chaimani A, Higgins JP, Mavridis D, Spyridonos P, Salanti G (2013). Graphical tools for network meta-analysis in STATA. PLoS ONE.

[15] Higgins JP, Thompson SG, Deeks JJ, Altman DG (2003). Measuring inconsistency in meta-analyses. BMJ (Clinical research ed).

[16] Lu G, Ades AE (2004). Combination of direct and indirect evidence in mixed treatment comparisons. Stat Med.

[17] Gelman BA (1998). General Methods for Monitoring Convergence of Iterative Simulations. J Comput Graph Stat.

[18] Salanti G, Ades AE, Ioannidis JP (2011). Graphical methods and numerical summaries for presenting results from multiple-treatment meta-analysis: an overview and tutorial. J Clin Epidemiol.

[19] Park K, Tan EH, O'Byrne K (2016). Afatinib versus gefitinib as first-line treatment of patients with EGFR mutation-positive non-small-cell lung cancer (LUX-Lung 7): a phase 2B, open-label, randomised controlled trial. Lancet Oncol.

[20] Paz-Ares L, Tan EH, O'Byrne K (2017). Afatinib versus gefitinib in patients with EGFR mutation-positive advanced non-small-cell lung cancer: overall survival data from the phase IIb LUX-Lung 7 trial. Ann Oncol.

[21] Hosomi Y, Morita S, Sugawara S (2020). Gefitinib Alone Versus Gefitinib Plus Chemotherapy for Non-Small-Cell Lung Cancer With Mutated Epidermal Growth Factor Receptor: NEJ009 Study. J Clin Oncol.

[22] Saito H, Fukuhara T, Furuya N (2019). Erlotinib plus bevacizumab versus erlotinib alone in patients with EGFR-positive advanced non-squamous non-small-cell lung cancer (NEJ026): interim analysis of an open-label, randomised, multicentre, phase 3 trial. Lancet Oncol.

[23] Zhao Y, Liu J, Cai X (2019). Efficacy and safety of first line treatments for patients with advanced epidermal growth factor receptor mutated, non-small cell lung cancer: systematic review and network meta-analysis. BMJ (Clinical research ed).

[24] Ramalingam SS, Vansteenkiste J, Planchard D (2020). Overall Survival with Osimertinib in Untreated, EGFR-Mutated Advanced NSCLC. N Engl J Med.

[25] Cross DA, Ashton SE, Ghiorghiu S (2014). AZD9291, an irreversible EGFR TKI, overcomes T790M-mediated resistance to EGFR inhibitors in lung cancer. Cancer Discov.

[26] Oxnard GR, Arcila ME, Sima CS (2011). Acquired resistance to EGFR tyrosine kinase inhibitors in EGFR-mutant lung cancer: distinct natural history of patients with tumors harboring the T790M mutation. Clin Cancer Res.

[27] Yu HA, Arcila ME, Rekhtman N (2013). Analysis of tumor specimens at the time of acquired resistance to EGFR-TKI therapy in 155 patients with EGFR-mutant lung cancers. Clin Cancer Res.

[28] Bearz A, Fratino L, Spazzapan S (2007). Gefitinib in the treatment of elderly patients with advanced non-small cell lung cancer (NSCLC). Lung cancer (Amsterdam, Netherlands).

[29] Liu S, Wang D, Chen B, Wang Y, Zhao W, Wu J (2011). The safety and efficacy of EGFR TKIs monotherapy versus single-agent chemotherapy using third-generation cytotoxics as the first-line treatment for patients with advanced non-small cell lung cancer and poor performance status. Lung Cancer (Amsterdam, Netherlands).

[30] Sequist LV, Yang JC, Yamamoto N (2013). Phase III study of afatinib or cisplatin plus pemetrexed in patients with metastatic lung adenocarcinoma with EGFR mutations. J Clin Oncol.

[31] Cheng Y, Murakami H, Yang PC (2016). Randomized Phase II Trial of Gefitinib With and Without Pemetrexed as First-Line Therapy in Patients With Advanced Nonsquamous Non-Small-Cell Lung Cancer With Activating Epidermal Growth Factor Receptor Mutations. J Clin Oncol.

[32] Yang JC, Cheng Y, Murakami H (2020). A Randomized Phase 2 Study of Gefitinib With or Without Pemetrexed as First-line Treatment in Nonsquamous NSCLC With EGFR Mutation: Final Overall Survival and Biomarker Analysis. J Thoracic Oncol.

[33] Chen CY, Chang YL, Shih JY (2011). Thymidylate synthase and dihydrofolate reductase expression in non-small cell lung carcinoma: the association with treatment efficacy of pemetrexed. Lung cancer (Amsterdam, Netherlands).

[34] Yang CH, Huang CJ, Yang CS (2005). Gefitinib reverses chemotherapy resistance in gefitinib-insensitive multidrug resistant cancer cells expressing ATP-binding cassette family protein. Can Res.

