# Supplementary material for: Efficacy of first-line treatments in the elderly and non-elderly patients with advanced epidermal growth factor receptor mutated, non-small cell lung cancer: a network meta-analysis
Source: BMC Cancer. 2022 May 7;22:514. doi: 10.1186/s12885-022-09592-3 (PMC9077975; doi:10.1186/s12885-022-09592-3)

**Table S1 Literature search criteria for PubMed**

| (((((((((((((((((Carcinoma, Non-Small-Cell Lung[MeSH Terms]) OR (non-small-cell lung cancer[Title/Abstract])) OR (non-small cell lung cancer[Title/Abstract])) OR (non small-cell lung cancer[Title/Abstract])) OR (non small cell lung cancer[Title/Abstract])) OR (non-small-cell lung carcinoma[Title/Abstract])) OR (non-small cell lung carcinoma[Title/Abstract])) OR (non small-cell lung carcinoma[Title/Abstract])) OR (non small cell lung carcinoma[Title/Abstract])) OR (nsclc[Title/Abstract])) OR (lung non-squamous[Title/Abstract])) OR (lung nonsquamous[Title/Abstract])) OR (lung adenocarcinoma[Title/Abstract])) AND ((epidermal growth factor receptor[Title/Abstract]) OR (EGFR[Title/Abstract]))) AND (((((((((((treatment[Title/Abstract]) OR (therapy[Title/Abstract])) OR (tyrosine kinase inhibitor[Title/Abstract])) OR (TKI[Title/Abstract])) OR (osimertinib[Title/Abstract])) OR (dacomitinib[Title/Abstract])) OR (afatinib[Title/Abstract])) OR (erlotinib[Title/Abstract])) OR (gefitinib[Title/Abstract])) OR (icotinib[Title/Abstract])) OR (chemotherapy[Title/Abstract]) OR ((((first-line[Title/Abstract]) OR (first line[Title/Abstract])) OR (treatment-naive[Title/Abstract])) OR (treatment-naïve[Title/Abstract])) OR (untreated[Title/Abstract]))) AND ((((((((OS[Title/Abstract]) OR (overall survival[Title/Abstract])) OR (overall-survival[Title/Abstract]) ) OR (MST[Title/Abstract])) OR (progression-free survival[Title/Abstract])) OR (progression free survival[Title/Abstract])) OR (PFS[Title/Abstract])) OR (iPFS[Title/Abstract]))) AND (((((((Randomized Controlled Trial[Publication Type]) OR (controlled clinical trial[Publication Type])) OR (randomized[Title/Abstract])) OR (randomised[Title/Abstract])) OR (randomly[Title/Abstract])) OR (trial*[Title/Abstract])) OR (phase[Title/Abstract]))) AND (("0001/01/01"[Date - Publication] : "2020/09/30"[Date - Publication])) |
| --- |

**Table S2 Literature search criteria for Embase**

| ('lung tumor'/exp OR 'non-small-cell lung cancer':ti OR 'non-small cell lung cancer':ti OR 'non small-cell lung cancer':ti OR 'non small cell lung cancer':ti OR 'non-small-cell lung carcinoma':ti OR 'non-small cell lung carcinoma':ti OR 'non small-cell lung carcinoma':ti OR 'non small cell lung carcinoma':ti OR 'nsclc':ti OR 'lung non-squamous':ti OR 'lung nonsquamous':ti OR 'lung adenocarcinoma':ti) AND ('epidermal growth factor receptor':ab,ti OR 'EGFR':ab,ti ) AND ('treatment':ab,ti OR 'therapy':ab,ti OR 'tyrosine kinase inhibitor':ab,ti OR 'TKI':ab,ti OR 'osimertinib':ab,ti OR 'dacomitinib':ab,ti OR 'afatinib':ab,ti OR 'erlotinib':ab,ti OR 'gefitinib':ab,ti OR 'icotinib':ab,ti OR 'chemotherapy':ab,ti OR 'first-line':ab,ti OR 'first line':ab,ti OR 'treatment-naive':ab,ti OR 'treatment-naïve':ab,ti OR 'untreated':ab,ti ) AND ('OS':ab,ti OR 'overall survival':ab,ti OR 'overall-survival':ab,ti OR 'MST':ab,ti OR 'progression-free survival':ab,ti OR 'progression free survival':ab,ti OR 'PFS':ab,ti) AND ('Randomized Controlled Trial':it OR 'controlled clinical trial':it OR 'randomized':ab,ti OR 'randomised':ab,ti OR 'randomly':ab,ti OR 'trial':ab,ti OR 'phase':ab,ti) AND [1966-2020]/py |
| --- |

**Table S3 Literature search criteria for Cochrane**

| ((non-small-cell lung cancer):ti OR (non-small cell lung cancer):ti OR (non small-cell lung cancer):ti OR (non small cell lung cancer):ti OR (non-small-cell lung carcinoma):ti OR (non-small cell lung carcinoma):ti OR (non small-cell lung carcinoma):ti OR (non small cell lung carcinoma):ti OR (nsclc):ti OR (lung non-squamous):ti OR (lung nonsquamous):ti OR (lung adenocarcinoma):ti) AND ((epidermal growth factor receptor):ti,ab,kw OR (EGFR):ti,ab,kw ) AND ((treatment):ti,ab,kw OR (therapy):ti,ab,kw OR (tyrosine kinase inhibitor):ti,ab,kw OR (TKI):ti,ab,kw OR (osimertinib):ti,ab,kw OR (dacomitinib):ti,ab,kw OR (afatinib):ti,ab,kw OR (erlotinib):ti,ab,kw OR (gefitinib):ti,ab,kw OR (icotinib):ti,ab,kw OR (chemotherapy):ti,ab,kw OR (first-line):ti,ab,kw OR (first line):ti,ab,kw OR (treatment-naive):ti,ab,kw OR (treatment-naïve):ti,ab,kw OR (untreated):ti,ab,kw ) AND ((OS):ti,ab,kw OR (overall survival):ti,ab,kw OR (overall-survival):ti,ab,kw OR (MST):ti,ab,kw OR (progression-free survival):ti,ab,kw OR (progression free survival):ti,ab,kw OR (PFS):ti,ab,kw) AND ((Randomized Controlled Trial):pt OR (controlled clinical trial):pt OR (randomized):ti,ab,kw OR (randomised):ti,ab,kw OR (randomly):ti,ab,kw OR (trial):ti,ab,kw OR (phase):ti,ab,kw) |
| --- |

**Table S4 Comparisions of the fit of consistency and inconsistency models using deviance information criteria (DIC)**

| Model | PFS | | |  | OS | | |
| --- | --- | --- | --- | --- | --- | --- | --- |
|  | All | < 65 years old | >= 65 years old |  | All | < 65 years old | >= 65 years old |
| Consistency | 43.171 | 23.992 | 23.851 |  | 23.946 | 12.597 | 23.851 |
| Inconsistency | 41.634 | 23.802 | 24.082 |  | 24.325 | 14.038 | 24.082 |

PFS: progression free survival, OS: overall survival.

The DIC is a Bayesian model evaluation criteria that measures model fit adjusted with complexity of the model^1^.

Smaller DIC value correspond to more preferable models. Difference between DIC from different models below 5 could be accepted.

References:

1. Spiegelhalter DJ, Best NG, Linde C. Bayesian measures of model complexity and fit. Journal of the Royal Statistical Society: Series B (Statistical Methodology) 2002;64:583-639.

**Table S5 Node-splitting analysis of inconsistency.**

**All P value was below 0.05, indicating no significant inconsistencies between the direct effect and indirect effects.**

| **Nodes** | **Direct effect** | **Indirect effect** | **Overall** | **P** |
| --- | --- | --- | --- | --- |
| **PFS: Overall** |  |  |  |  |
| Chemotherapy, Afatinib | 2.30(1.70-3.20) | 5.00(2.90-8.40) | 2.90(2.10-4.10) | 0.025 |
| SoC, Afatinib | 1.30(0.90-2.00) | 0.61(0.39-1.00) | 0.97(0.66-1.40) | 0.022 |
| SoC, Chemotherapy | 0.27(0.18-0.38) | 0.57(0.34-0.92) | 0.34(0.23-0.47) | 0.020 |
| **PFS: <65 years old** |  |  |  |  |
| Chemotherapy, Afatinib | 2.30(0.95-5.70) | 5.20(1.10-27.00) | 2.80(1.30-6.20) | 0.245 |
| SoC, Afatinib | 1.50(0.40-5.20) | 0.65(0.17-2.50) | 1.00(0.39-2.60) | 0.235 |
| SoC, Chemotherapy | 0.29(0.11-0.81) | 0.64(0.12-0.36) | 0.36(0.15-0.84) | 0.264 |
| **PFS: >= 65 years old** |  |  |  |  |
| Chemotherapy, Afatinib | 2.70(0.78-11.00) | 5.20(0.56-49.00) | 3.20(1.30-10.00) | 0.482 |
| SoC, Afatinib | 1.20(0.18-7.30) | 0.60(0.10-4.00) | 0.91(0.27-3.00) | 0.495 |
| SoC, Chemotherapy | 0.23(0.06-0.79) | 0.45(0.04-3.50) | 0.28(0.09-0.76) | 0.488 |
|  |  |  |  |  |
| **OS: Overall** |  |  |  |  |
| Chemotherapy, Afatinib | 1.30(0.97-1.70) | 0.92(0.50-1.70) | 1.20(0.94-1.60) | 0.288 |
| SoC, Afatinib | 1.10(0.76-1.70) | 1.60(0.89-2.80) | 1.30(0.94-1.70) | 0.329 |
| SoC, Chemotherapy | 1.20(0.79-2.00) | 0.90(0.55-1.40) | 1.00(0.76-1.50) | 0.356 |
| **OS: <65 years old** |  |  |  |  |
| Chemotherapy, Afatinib | 1.20(0.69-2.00) | 1.30(0.42-4.40) | 1.20(0.82-1.80) | 0.758 |
| SoC, Afatinib | 1.50(0.69-3.40) | 1.30(0.49-3.70) | 1.40(0.88-2.30) | 0.795 |
| SoC, Chemotherapy | 1.10(0.51-2.40) | 1.30(0.51-3.20) | 1.20(0.72-2.00) | 0.779 |
| **OS: >= 65 years old** |  |  |  |  |
| Chemotherapy, Afatinib | 2.70(0.78-11.00) | 5.20(0.56-49.00) | 3.20(1.30-10.00) | 0.482 |
| SoC, Afatinib | 1.20(0.18-7.30) | 0.60(0.10-4.10) | 0.91(0.27-3.00) | 0.495 |
| SoC, Chemotherapy | 0.23(0.06-0.79) | 0.45(0.04-3.50) | 0.28(0.09-0.76) | 0.488 |

PFS: progression free survival, OS: overall survival.

**Figure S1 Density plot for progression-free survival (PFS) and overall survival (OS) in all included patients, in elderly patients and in non-elderly patients.**

1.
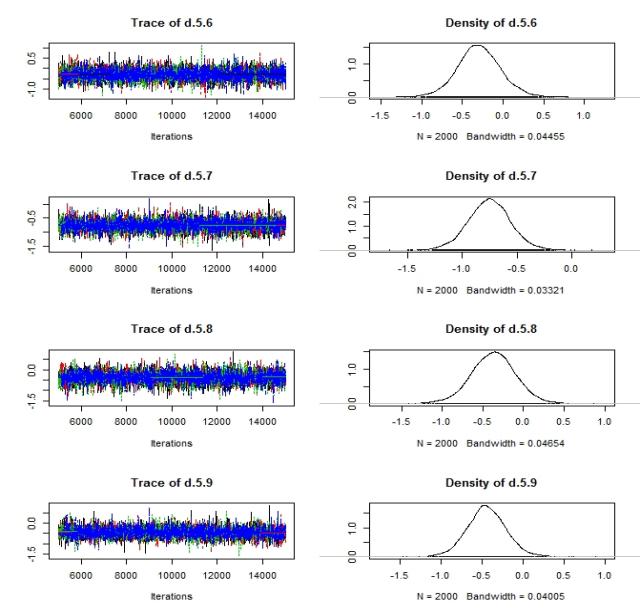
 Density plot for PFS in all patients


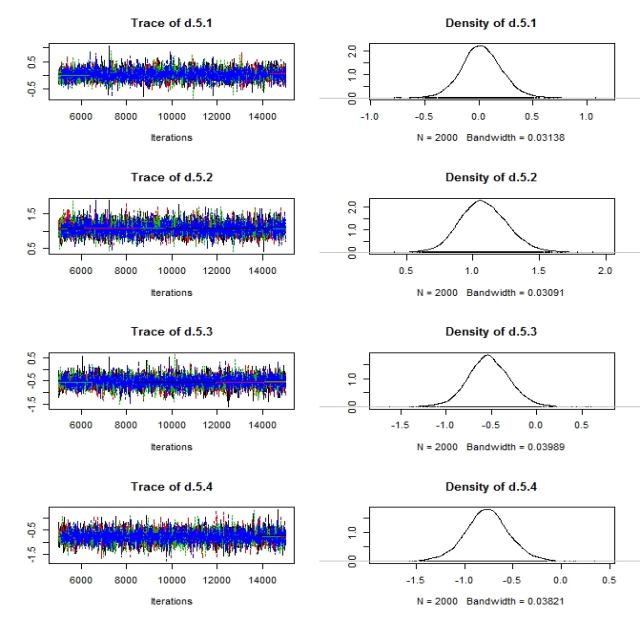


B)Density plot for PFS in patients below 65 years old


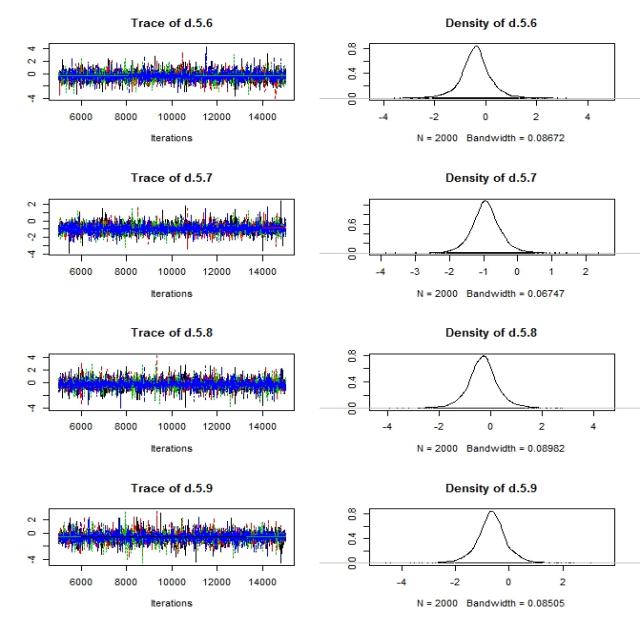

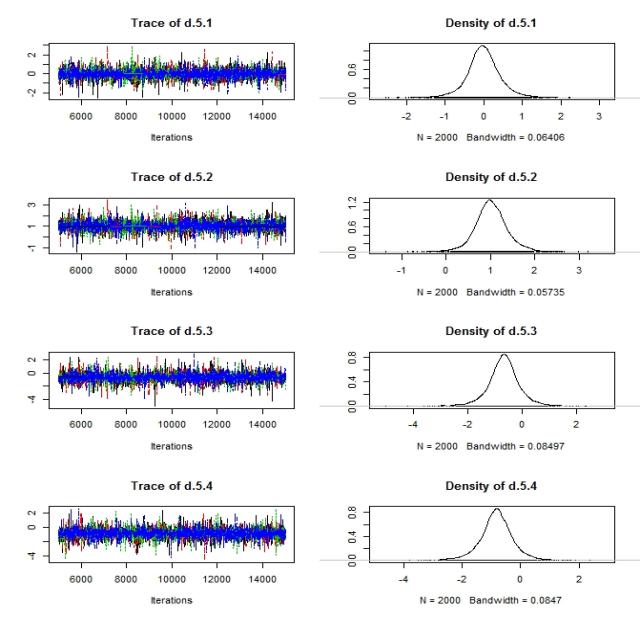


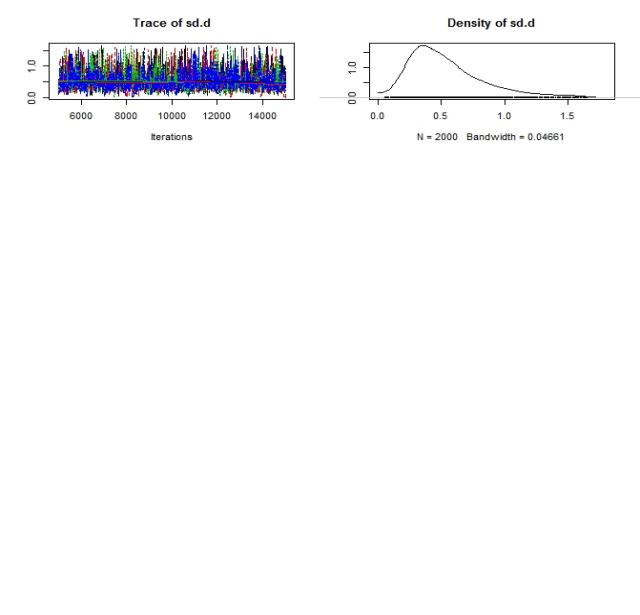


C) Density plot for PFS in patients over 65 years old


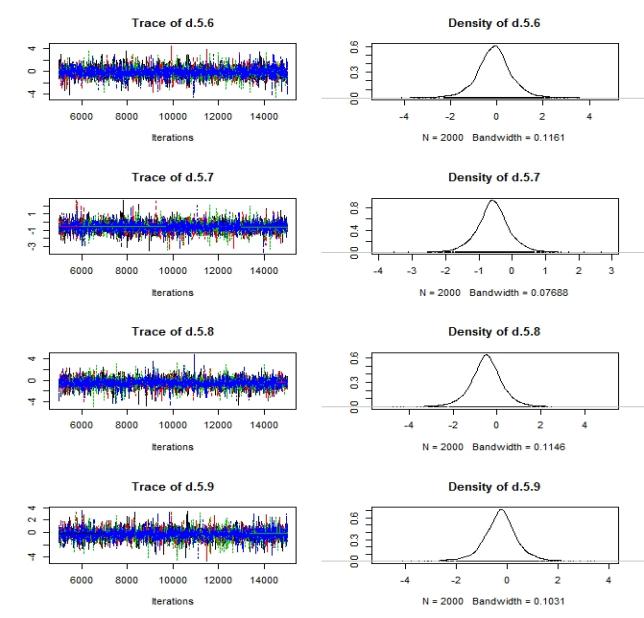

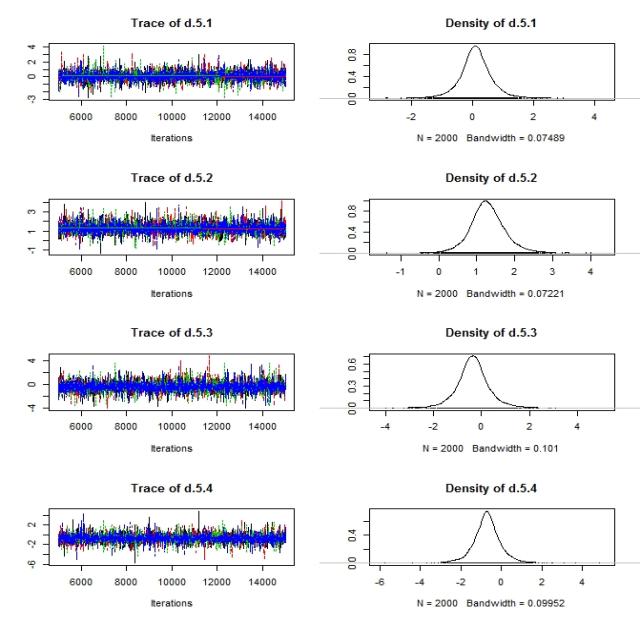


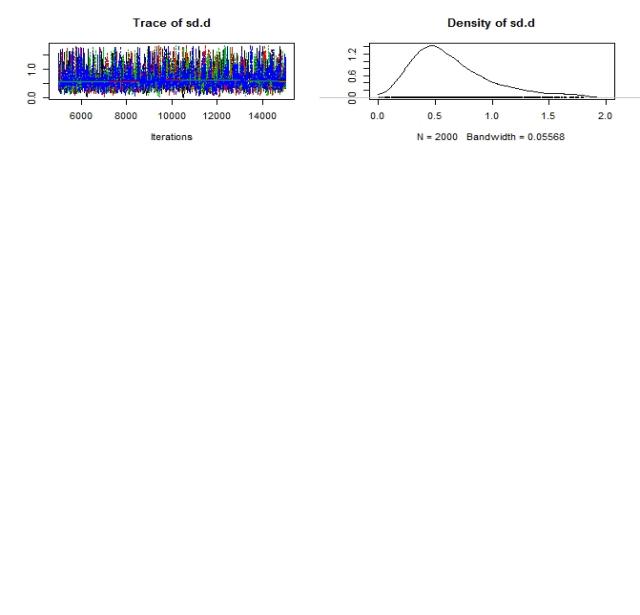


D) Density plot for OS in all patients


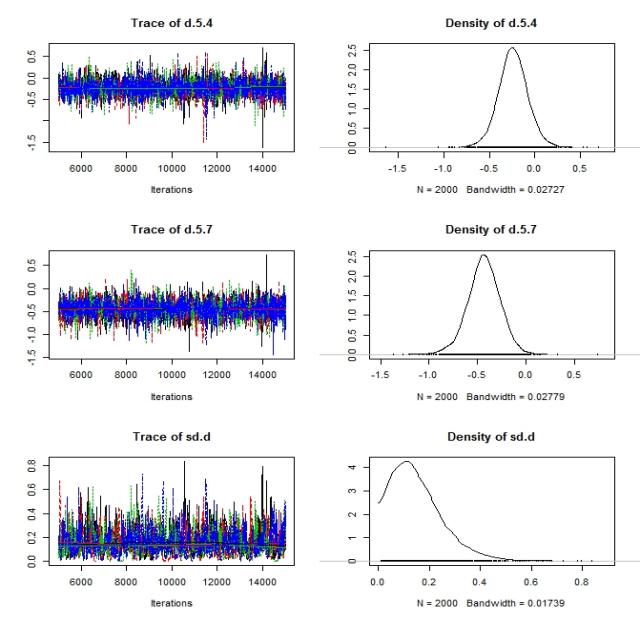

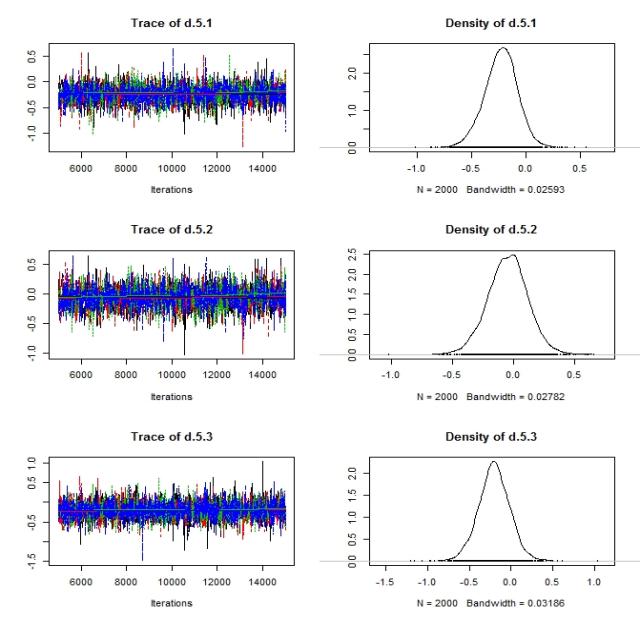


E) Density plot for OS in patients below 65 years old


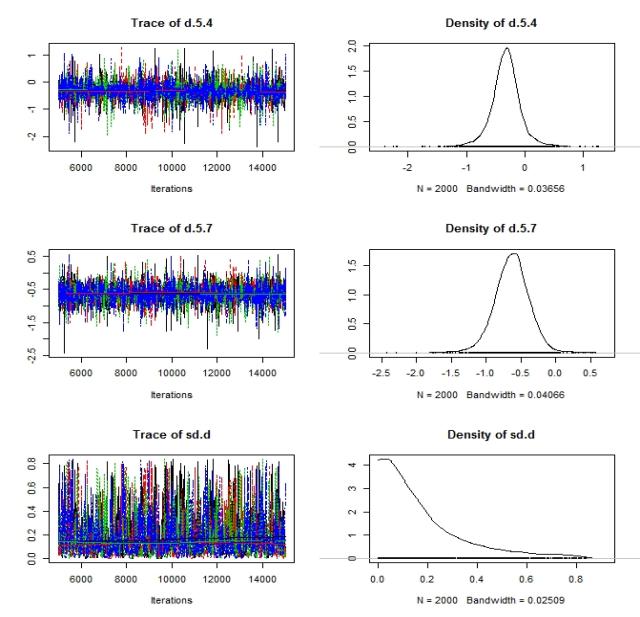

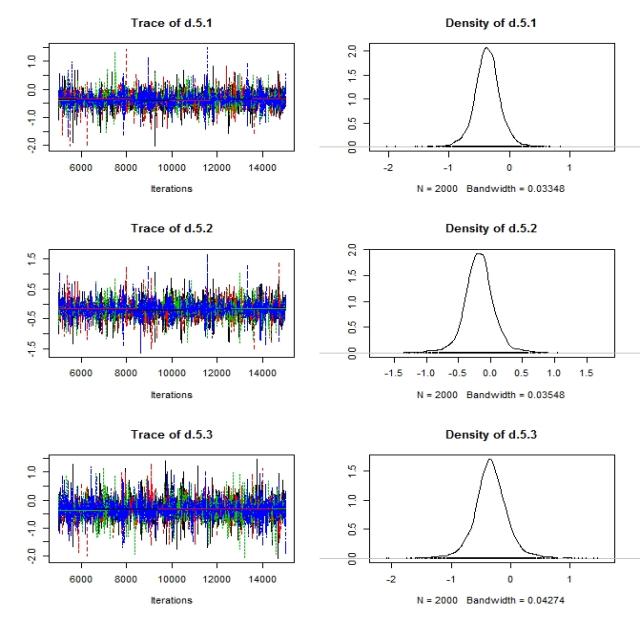


F) Density plot for OS in patients over 65 years old


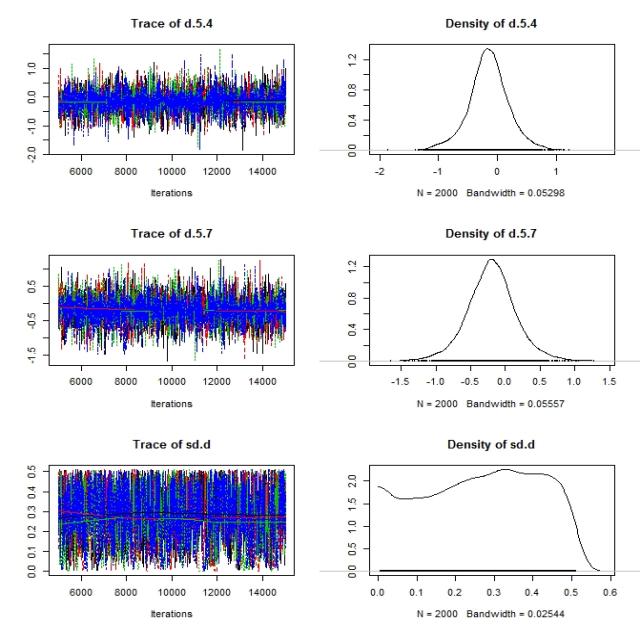

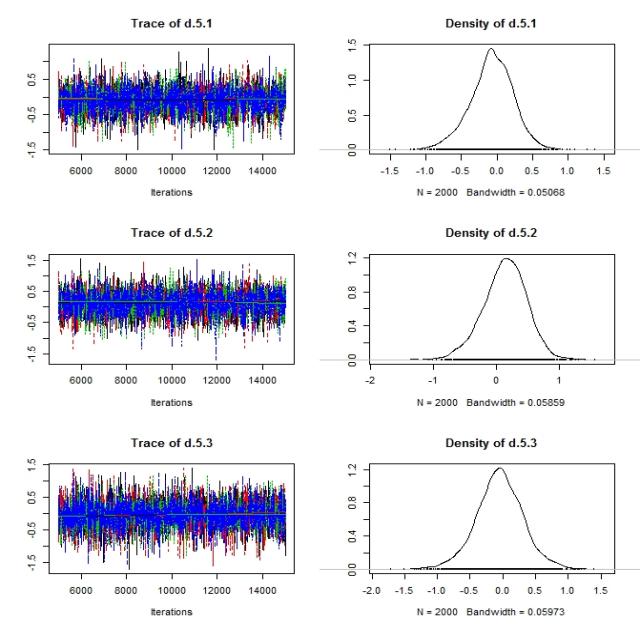

Supplement: Supplementary file 1 — Additional file 1: Table S1. Literature search criteria for PubMed. Table S2. Literature search criteria for Embase. Table S3. Literature search criteria for Cochrane. Table S4. Comparisions of the fit ofconsistency and inconsistency models using deviance information criteria (DIC). Table S5. Node-splitting analysis ofinconsistency.All P value was below 0.05, indicatingno significant inconsistencies between the direct effect and indirect effects. Figure S1. Density plot forprogression-free survival (PFS) and overall survival (OS) in all includedpatients, in elderly patients and in non-elderly patients. [file 12885_2022_9592_MOESM1_ESM.docx]
